# Influence of Post-Harvest 1-Methylcyclopropene (1-MCP) Treatment and Refrigeration on Chemical Composition, Phenolic Profile and Antioxidant Modifications during Storage of Abate Fétel Pears

**DOI:** 10.3390/antiox12111955

**Published:** 2023-11-02

**Authors:** Paola Tedeschi, Silvia Marzocchi, Nicola Marchetti, Francisco J. Barba, Annalisa Maietti

**Affiliations:** 1 Department of Chemical, Pharmaceutical and Agricultural Sciences, University of Ferrara, 44121 Ferrara, Italy; nicola.marchetti@unife.it (N.M.); annalisa.maietti@unife.it (A.M.); 2 Department of Agricultural and Food Sciences, University of Bologna, 47521 Cesena, Italy; silvia.marzocchi4@unibo.it; 3 Nutrition, Food Science and Toxicology Department, Faculty of Pharmacy, University of València, Avda. Vicent Andrés Estellés, s/n, Burjassot, 46100 València, Spain; francisco.barba@uv.es

**Keywords:** Abate Fétel pear, shelf-life, 1-MCP, chemical composition, phenolic compound, antioxidant activity

## Abstract

‘Abate Fétel’, a winter cultivar, is the most important pear cultivar in Italy; its fruits are appreciated by consumers for their aroma, texture and balanced sweet and sour taste. Maintaining high-quality characteristics to prolong the shelf-life of fruit and preserve the sensory and nutritional quality is a priority for the food industry. The aim of our study was to test the effectiveness of 1-methylcyclopropene (1-MCP) and cold storage in prolonging the shelf-life of these fruits, which were harvested at maturity at two different times. This work focused on the effects of different storage treatments and two ripening times on (i) the chemical composition of Abate Fétel pulp fruits to preserve their sweet taste and aroma and (ii) the phenolic profile composition and antioxidant activity of the peel, which is naturally rich in phytochemicals and important for the fruit’s shelf-life and in the functional food industry for its high nutritional value. Abate Fétel fruits were harvested at the optimal commercial maturity stage, first on 15 September, having been treated with 1-MCP and stored for 2 months at cold temperatures; the other fruits were harvested at the end of September and stored in a cold cell for 2 months. The fruit pulp was tested for glucose and fructose, pH, acidity and organic acids (malic, citric, fumaric and shikimic), phenolic content and phenolic compounds (chlorogenic and caffeic acids, rutin, hyperoside, kaempferol-3-rutinoside and isoquercitrin), and the antioxidant activities in the fruit peels were measured. Treating the fruits with 1-MCP better preserved the phytochemical compounds compared to simple refrigeration, preserving the fruit’s quality and prolonging its shelf-life. All the treatments help to maintain the glucose and fructose content and the acidity, preserving the aroma and organoleptic characteristics.

## 1. Introduction

Pear fruits (*Pyrus communis)* are particularly appreciated by consumers for their quality features, such as their unique shape, buttery texture, juiciness, sweetness and aroma. Italy is the fourth highest producer of pears, after China, Argentina and the United States [1].

The amount of Italian pears destined for export is about 20% of the total produced, with an increasing trend. Almost 100,000 of the more than 150,000 tons of exported pears come from the Emilia Romagna region. More than 90% of exports are destined for the European Union (EU), especially Germany, which imports half of the total, followed by France (18%), the United Kingdom (8%) and Austria (5%). Other minor amounts of this product are destined for outside the EU, in particular Russia, the USA, Switzerland, and such eastern European countries as Croatia.

In 1998, Emilia Romagna pears obtained protected geographical indication (PGI) status from the European Union, of which Abate Fétel are the most prized of all, which are picked in September and are only grown in the Emilia area. Other varieties, for example, the Conference pear, the Decana del Comizio pear, the Kaiser pear, the Max Red Bartlett pear and the Williams pear, all with distinctive qualities, are grown in the provinces of Ferrara, Modena, Reggio Emilia, Bologna and Ravenna.

Originating in France and named “Abbè Fétel” for an abbot who discovered it in the mid-1800s, Abate Fétel pears are large (up to 260 g) fruits, characterized by a thin, yellowish and slightly rusty skin around the stalk, sometimes saturated with red on the side most exposed to the sun, a white juicy pulp and a sweet–sour taste. In Emilia Romagna, Abate Fétel is the main cultivated variety (around 42%) and is among the most popular for fresh consumption.

Pear fruits are in high demand because they are rich in nutrients, fiber, sugars (which confer sweetness), organic acids (that provide fruit sourness) and other bioactive compounds that offer beneficial effects to health [2,3]. These compounds, in particular phenolic compounds, are a crucial constituent of the human diet due to their important antioxidant activity and related disease prevention abilities [4]. Moreover, natural antioxidants, especially plant-derived antioxidants, are preferred over synthetic antioxidants in food and food supplement industries, so the importance of the levels of these compounds in fruits has become much more important [4,5,6].

Pears and some fresh fruits continue to ripen after harvest, and this process not only speeds up product maturation but also affects their texture, aroma and composition and shortens their shelf-life [7,8].

After harvesting, pears are on sale until the following spring, stored in a controlled atmosphere or routinely cold-stored to guarantee long-term availability [9]. It is widely known that ethylene is involved in the ripening of climacteric fruit, among which are pears, promoting important fruit characteristics such as texture, flavor, color and nutritional and functional properties. Nevertheless, ethylene can also induce the development of physiological disorders, senescence and increased pathogen susceptibility, reducing the shelf-life [10].

Treatments to protect fruit quality and prolong shelf-life are increasingly sought after, and 1-methylcyclopropene (1-MCP) is considered an effective inhibitor of ethylene production, binding to the copper metal present in the ethylene receptors and thus blocking the normal system of ethylene production as well as system II. Accumulation of 1-aminocyclopropane-1-carboxilate synthase and oxidase (ACC-synthase and ACC-oxidase, two enzymes involved in ethylene production) is reduced by 1-MCP, decreasing ethylene production [11,12].

As a result, 1-MCP is effective in maintaining fruit quality, reducing chilling injuries during the storage period and extending the shelf-life of some climacteric and non-climacteric fruit [13,14,15,16,17,18]. Furthermore, Xu et al. [16] and Zhang et al. [8], in previous studies, demonstrated a positive effect of 1-MCP on the quality and antioxidant enzyme activity, correlating 1-MCP treatment with the expression of genes related to phenolic metabolism.

Specifically, 1-MCP induced the activities of some antioxidant enzymes (superoxide dismutase, catalase and peroxidase), which are very important in scavenging ROS to protect cell membranes, delay fruit senescence and improve shelf-life [19].

A gaseous molecule, 1-MCP is easy to apply and has an excellent safety profile, without causing residual effects in treated produce. Thus, 1-MCP use has been granted approval by regulatory bodies in more than 40 countries for a wide variety of fruits and vegetables [11].

AgroFresh Inc, under the trade name SmartFresh, uses 1-MCP on edible crops. SmartFresh^TM^ is a sugar-based powder, which dissolves in tap water and releases 1-MCP in storage. It permeates the air and interacts with ethylene receptors, maintaining important fruit characteristics [20]. Post-harvest application of 1-MCP, leading to a decreased softening of Bartlett, Williams, Passe-Crassane and Conference pears, was studied in [21,22,23,24], but very few studies can be found on Abate Fétel pears.

Moreover, texture and sweetness are major attributes that have a strong effect on consumer perceptions of pear fruit quality, and various studies have underlined the importance of 1-MCP on reducing senescence, internal browning, superficial scald symptoms and flesh firmness. However, very few authors have focused their attention on the functional components of pear peel and the effect of 1-MCP on its antioxidant compounds [25]. Pears, like other plants, produce secondary metabolites under stress conditions, and phenolic compounds are an important secondary metabolite group produced during plant responses to challenging conditions [4]. Polyphenols are considered powerful antioxidants, studied for their potential to protect the human body against oxidative stress, but are also important for maintaining nutritional quality and extending products’ shelf-lives [26]. Since most phenolics are located in the peel, it is important to protect them and keep these metabolites in good condition and maintain the fruit nutritional value during the post-harvest period.

This study aimed to compare two storage conditions of Abate Fétel pears harvested at two different times in the Ferrara province: cold-storage and cold-storage combined with 1-MCP treatment. Research on changes in organic acid and polyphenol contents during the shelf-life of fruits is limited, and the aim of our work is (i) to evaluate the fruit conservation process with respect to the harvest period, (ii) to investigate the effect of 1-MCP and cold storage treatments on the sugar, acidity and organic acid contents of Abate Fétel pulp to preserve its sweet taste and aroma and (iii) to evaluate the effect of different storage treatments on the phenolic composition and antioxidant activity of the peel, which is naturally rich in phytochemicals and important for fruit shelf-life and in the functional foods industry for its high nutritional value.

## 2. Materials and Methods

### 2.1. Plant Material

The pear samples (Abate Fétel variety) analyzed in this study came from a company in the province of Ferrara. Fruits were harvested at the optimal commercial maturity stage at two sampling times (15 September—SF and 30 September—CS), and 15 pears from each harvest were transported to our university (Time T0).

The pears picked on 15 September were treated with 20 mg/m^3^ of 1-methylcyclopropene (SmartFresh^®^, AgroFresh, Milan, Italy) for 24 h according to the manufacturer’s recommendations and stored at 1 ± 2 °C in a climatic cell. The pears picked on 30 September were simply stored in a refrigerated cell (1 °C) owned by the collection company in Ferrara (Italy). At the end of November, 60 fruits from each treatment (15 fruits per replication) were taken to our laboratory.

**Figure 1:**
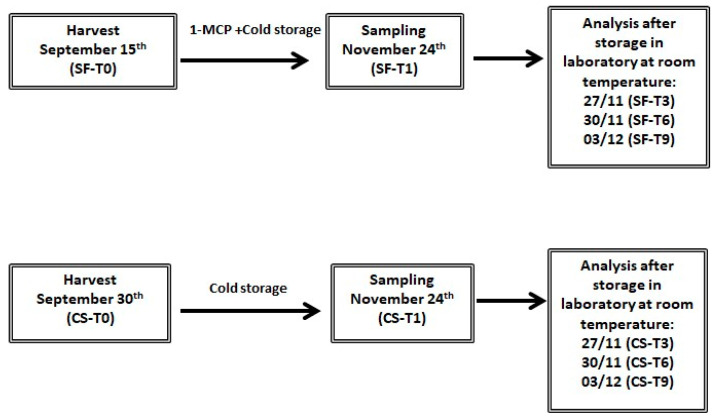
Harvest and storage treatments with relative acronyms.

Measurements were taken of fruit at harvest (control fruit without any treatment—T0), after treatment application (T1) and subsequently after maturation in the laboratory for 3 (T3), 6 (T6) and 9 (T9) days at room temperature (Figure 1). The fruit were peeled (skin thickness approximately 1 mm) and cut (cube size 1 cm) before analyses.

### 2.2. Glucose and Fructose Quantification

Sugar determination (glucose and fructose) was performed using an enzymatic kit (Glucose and Fructose Assay Kit, Megazyme, Astori Tecnica, Poncarale, BS, Italy). An amount of 5 g of pear pulp was added to 50 mL of water, homogenized with Ultra-Turrax and centrifuged at 9000× *g* for 10 min. The supernatant was collected and suitably diluted (1:20) with distilled water. Water (2 mL), 0.1 mL of sample, 0.1 mL of buffer and 0.1 mL of solution containing NADP+ and ATP were added to each cuvette. Sugar analyses were performed spectrophotometrically by reading the absorbance at λ = 340 nm. After 3 min, the absorbance was read (A1). A hexokinase (HK) and glucose-6-phosphate dehydrogenase (G6P-DH) solution (20 µL) was added to the mixture, and after 5 min, the absorbance was read again (A2). Finally, 0.02 mL of phosphoglucose isomerase (PGI) was added, and the final absorbance (A3) was measured.

Glucose and fructose (g/L) were calculated as

C = [(V × MW)/(ε × D × v)] × ΔA

where

V = final volume; MW = molecular weight of glucose or fructose (g/mol);

ε = 6300 (L·mol^−1^·cm^−1^); molar coefficient of extinction for NADPH at 340 nm;

D = 1 cm;

v = sample volume;

∆A = (A2 − A1) for glucose and (A3 − A2) for fructose quantification.

Glucose and fructose were then expressed as g/100 g of fresh matter.

### 2.3. Determination of pH and Titratable Acidity

Pear fruit pulp was ground in a Retsch GM200 mill (Retsch Verder Scientific S.r.l., Torre Boldone, Bergamo, Italy) at 3000× *g* for 30 s, and 10 g of blended pulp was suspended in water up to a volume of 150 mL. The pH of the homogenized pear was measured and the acidity was determined by titration with 0.1 mol L^−1^ NaOH up to 8.1 according to the literature [27]. Titratable acidity was expressed as g of malic acid eq./100 g of fresh pear fruit. Each sample was titrated in triplicate.

### 2.4. Determination of Organic Acid by Capillary Electrophoresis

An amount of 30 g of pear pulp was added to 30 mL of MilliQ water and blended. The homogenate was centrifuged for 5 min at 10,100× *g* (Centrifuge Thermo PK121R, Thermo Fisher Scientific, Waltham, MA, USA); then, the supernatant was filtered with a membrane filter (Minisart RC 25 filter, 0.45 µm). The organic acid readings were performed by CE Beckman P/ACE^TM^ MDQ with a diode array detector (Beckman Coulter, Milan, Italy). Fumaric acid, malic acid, citric acid and shikimic acid standards were prepared at different concentrations. For fumaric acid, the calibration curve was 0.5–6 µg/mL (R^2^ = 0.9959), for malic acid it was 50–600 µg/mL (R^2^ = 0.9948), for citric acid it was 5–60 µg/mL (R^2^ = 0.9956), and for shikimic acid it was 5–60 µg/mL (R^2^ = 0.9900).

Separation was performed by a 75 mm i.d. fused silica capillary with a total length of 50 cm maintained in a cartridge with a detector window of 190 mm × 250 mm. The capillary was conditioned before use by flushing with 0.1 M NaOH for 2 min, with water for 2 min and then with buffer for 3 min.

The buffer was composed of 180 mM Na_2_HPO_4_ and 1 mM CTAB, Ph = 6.25, with H_3_PO_4._ The sample was injected into the capillary by pressure injection for 5 s. Separation was conducted at −12 kV and 25 °C for 15 min. Data collection was achieved using P/ACE ^TM^ MDQ Station software (32 Karat Version 4.0).

### 2.5. Phenol Determination and Antioxidant Activity in Pear Skin Tissue

The extraction procedure was performed with 5 g of finely chopped pear skin with 15 mL of a methanol/water/formic acid 80/20/0.1% (*v*/*v*) solution at room temperature in the dark for 30 min under magnetic stirring. The sample was centrifuged (10.100× *g* at 4 °C for 5 min), and the supernatant was recovered. The extraction was repeated in sequence 3 times, and each supernatant was gathered and kept at −20 °C till analysis [28].

#### 2.5.1. HPLC/UV-Vis Analysis and Total Phenolic Content

A 1 mL aliquot of the extracted sample was filtered and injected into an HPLC machine. HPLC/UV–Vis analysis was performed on a Shimadzu VP Series (Shimadzu Italia, Milan, Italy) modular model system consisting of a vacuum degasser, a quaternary pump, an autosampler, a thermostatic column compartment and a UV–Vis detector. The analyses were carried out on a Kinetex C18 column (250 × 4.6 mm I.D., 5 µm, Phenomenex (Torrance, CA, USA)). The mobile phase was composed of (A) 0.1 M HCOOH in H_2_O and (B) ACN, and the gradient elution changed as follows: 0–25 min from 10% to 25% B, 25–26 min from 25% to 95% B, 26–30 min isocratic conditions. The post running time was 6 min. The flow rate was 0.8 mL/min, and the column temperature was set at 30 °C, while the injection volume was 10 µL. The chromatogram was integrated at 352 nm. Injection was performed in triplicate. Chlorogenic acid (R^2^ = 0.9998), caffeic acid (R^2^ = 0.9979), rutin (R^2^ = 0.999), hyperoside R^2^ = 0.9975), isoquercitrin (R^2^ = 0.9963) and kaempferol-3 rutinoside (R^2^ = 0.9988) were used as a standard for the calibration curve (1–50 µg/mL).

A partially modified Folin–Ciocalteu method described by Singleton et al. [29] was used to quantify the polyphenols. An amount of 500 µL of Folin-Ciocalteu reagent was added to 50 µL of pear skin tissue extract and left for 5 min in the dark at room temperature. Then, 2 mL of Na_2_CO_3_ was added up to a volume of 10 mL, and the solution was left for 90 min in the dark at room temperature. Measurements were performed using a Beckman DU730 UV–Vis spectrophotometer at 700 nm. The calibration curve was determined with gallic acid (0.5–10 µg/mL; R^2^ = 0.9974), and the total phenolics were expressed as gallic acid equivalents (mg of gallic acid equivalents/g of pear fresh peel).

#### 2.5.2. Antioxidant Activity (DPPH Scavenging Assay)

A DPPH scavenging assay was used to detect the antioxidant activity in pear skin tissue using a 2-diphenyl-1-picrylhydrazyl (DPPH) assay, according to the Fukumoto [30] method with minor modifications. The calibration curve was prepared with 0.05–1 mM Trolox^®^ (6-hydroxy-2,5,7,8-tetramethylchroman-2-carboxylic acid) in methanol (R^2^ = 0.9914). A deep purple DPPH 2,2-diphenyl-1-picrylhydrazyl solution (0.06 mM) was prepared in methanol, and the absorbance was measured at 515 nm using a Beckman DU730 UV–Vis spectrophotometer. Pear skin extract (50 µL) was added to 1450 µL of DPPH solution and kept for 15 min in the dark at room temperature. The decrease in spectrophotometric absorbance was registered, and the antioxidant activity was calculated by the percentage of inhibition of the DPPH radical. The data were expressed as mM of Trolox eq./g of fresh pear peel tissue.

### 2.6. Statistical Analysis

Data are expressed as the means ± standard deviation (SD). Differences between non-treated samples and treated samples were evaluated by a one-way ANOVA, and Tukey’s tests were used for means comparisons. Differences at *p* ≤ 0.05 were considered significant. All analyses were performed using the SPSS software package version 22.0 (SPSS Inc., Chicago, IL, USA).

## 3. Results and Discussion

Post-harvest storage of pears is an important strategy allowing the constant availability of fresh pear fruits on the market, guaranteeing and extending commercialization and meeting consumers’ needs [1].

Quality measured at the time of consumption allows for an overall assessment of cumulative effects on consumer acceptance. Abate Fétel pears can maintain acceptable eating quality at 20 °C for about 8–10 days, but Abate Fétel fruit are commonly harvested in September, with peak commercial distribution in November–December, thus highlighting the great importance of conservation treatment.

Fruits collected on 15 September and 30 September were maintained at room temperature (20 °C) for 15 days, after which, changing texture parameters, first of all firmness, were perceived at eating and not appreciated by the consumer. Market research has shown that consumers choose fruit based on appearance, but to purchase the product, some other factors are important and affect consumer choice, such as aroma, taste and flavor [3,31]. Additionally, in recent years, consumers have been very interested in the nutritional and health properties of the product.

### 3.1. Evaluation of Pulp Quality at Harvest and during Storage: Sugar, Acidity and Organic Acids

Pear fruits are widely used and appreciated throughout Italy and Europe; their sensory characteristics such as aroma, sweetness and acidity are probably the most important characteristics for consumers and for further transformation of the product into canned fruit [32,33,34]. Sugar contributes to sweetness, and in pear fruits, fructose dominates, followed by glucose (Table 1). Fructose and glucose increased over time from September to November, while no significant differences were observed during 9 days of storage for both refrigerated and 1-MCP-treated samples. The pH of fresh Abate Fétel pears remained constant from harvest till use over 9 days of storage; on the other hand, acidity, quantified using a titration method and expressed as malic acid equivalent, was reduced after SmartFresh^®^ treatment but presented a fluctuating trend in refrigerated samples (CS) (Table 1). The acidity of cold-stored samples was significantly lower than pears treated with 1-MCP, and this aspect can be correlated to the lower amount of acids (in particular malic acid, the most abundant one) at the second harvest point, 15 days after the first fruit collection. Neither the pH level nor sugars differed between the two untreated samples.

Moreover, the ratio of sugar to acid is commonly used to determine the sensory and flavor quality of fruit, and our data highlighted a higher ratio for fruits harvested at the end of September than samples collected on 15 September. For both harvest periods, the ratio in cold-stored pears and pears treated with 1-MCP was twice that of just-harvested fruits, and it was maintained during “homemade” storage (3, 6 and 9 days at room temperature), guaranteeing sweetness over time. However, in order to evaluate the effect of treatments and ripening on pear fruit quality, other compositional aspects such as organic acid content should be considered.

In this study, the effects of 1-MCP and cold storage on the organic acid composition of Abate Fétel pear fruits during the shelf-life period were examined (Table 2). The results showed that the treatments affected the fruit’s organic acid contents beyond the pear harvest period. Malic acid was predominant, in agreement with other data presented in the literature [35,36], followed by shikimic, citric and fumaric acid.

The highest amount of malic acid was detected in non-treated fruits at the first harvest on 15 September, probably due to lower fruit ripeness. Malic acid decreased after two months of storage with 1-MCP, but it was relatively constant after 9 days of room storage.

The pears collected at the end of September presented a significantly lower amount of malic acid than those harvested in the middle of September, and the concentration of malic acid did not change during cold room storage. As for the 1-MCP treatment, the malic acid content did not change during the 9 days of storage at room temperature; thus, after 2 months, the amount of malic acid was similar regardless of the treatment.

The citric acid content was not very high in pear fruits; it ranged from 21.60 µg/g to 52.40 µg/g. The trends in this acid were opposite to the previously described malic acid. The amount of citric acid was higher in fruits harvested at the end of September, with a decrease during the 2 months of storage at cold temperature and an increase during the 9 days at room temperature. The citric acid concentration in fruits harvested in the middle of September was lower than the fruits harvested at the end of September (34.49 ± 1.82 µg/g vs. 46.71 ± 0.86 µg/g). In addition, in fruits stored with 1-MCP, the citric acid content decreased after two months of storage, suggesting a possible catabolism of citrate at cold temperatures [37], which then increased during the 9 days of conservation at room temperature.

Fumaric acid was the organic acid present in the lowest concentration and showed little change after two months of storage with both treatments. However, during storage at room temperature, fumaric acid contents were highly decreased even after 3 days with 1-MCP treatment; this decrease was more gradual in refrigeration treatment. After 9 days of “home conservation”, the amount of fumaric acid was similar and not significantly different in both the storage treatments.

Shikimic acid is involved in the metabolic pathways used by bacteria, fungi, algae and plants for the biosynthesis of folates and aromatic amino acids. In plants, these aromatic amino acids are not only important components of protein biosynthesis, but they are also precursors for various secondary metabolites such as phenolic compounds [38]. In our study, the shikimic acid content did not decrease in two months of storage with 1-MPC but decreased during “home conservation” at room temperature even after 3 days (SF-T3). In comparison, the fruits harvested on 30 September and stored in a cold room exhibited a slightly lower shikimic acid concentration, which was maintained during cold storage and decreased only after 9 days at room temperature.

Total sugars and individual sugar combined with organic acids highly affect taste and organoleptic characteristics and quality of fruits [39]; the concentration of organic acids is usually high at the early stage of development of pear and then decreases in the late stage of fruit ripening [40]. In the measurements made during the shelf-life of our pear fruits, treated with 1-MCP and cold storage, malic and shikimic acids were maintained at high levels, after two months of storage, and the decrease in acidity during storage period may be attributed to increased utilization of malic acid during fruit ripening as indicated by Sahkale et al. [41].

### 3.2. Evaluation of Pear Peels: Phenolic Content and Antioxidant Activity

Nowadays, natural antioxidants are preferred over synthetic ones, and the increasing demand for natural antioxidants, especially plant-derived antioxidants, has made the evaluation of the antioxidant content in fruits much more important [5]. Phenolic compounds are secondary metabolites produced from plants under stress conditions and, as powerful antioxidants, are crucial regarding the shelf-life of the fruits; they delay fruit decay, have beneficial health effects and prevent many diseases [18,42]. These functional or nutraceutical foods are highly regarded by consumers because of their increasingly fast and stressful lifestyles. Therefore, it is important to protect phytochemicals such as polyphenols and their antioxidant activity in fruits after harvest. Abate Fétel fruits, and pears in general, are very important due to their good taste and low calorie and high fiber content but generally have lower phenolic compound content and antioxidant capacities in comparison with other fruits [43]. As described by other authors, pear pulp contains lower total polyphenol concentrations than the peel [36,44,45]; thus, our study focused on the phenolic composition in pear peels, evaluating the impacts of ripening and conservation on preservation of these important compounds.

As indicated in the literature [46,47], the total phenolic content depends on many different factors, such as pear cultivar, stage of maturity, post-harvest treatments and storage conditions. It is thus relevant to characterize and quantify antioxidants in pears during storage and evaluate the effect of storage conditions and different postharvest treatments on antioxidant composition.

**Figure 2:**
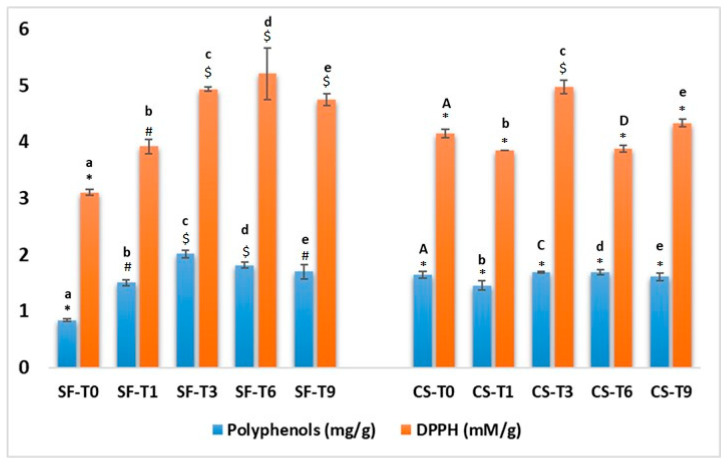
Total polyphenol content and antioxidant activity in Abate Fétel pear peels under different storage conditions, SmartFresh^®^ treatment (SF) and cold storage (CS), harvested at two different times. The results are expressed as means ± standard deviations. Different symbols indicate significant differences between samples of the same storage treatment. Lower- and uppercase letters indicate significant differences for SmartFresh (SF) samples vs. cold storage (CS) samples at the same sampling time (T0, T1, T3, T6, T9) (*p* ≤ 0.05).

After harvesting on 15 September, Abate Fétel pears exhibited the lowest polyphenol content (0.836 ± 0.024 mg of gallic acid eq./g of fresh peel), while after 2 months of storage with 1-MCP, the polyphenol content had increased to a value of 1.502 ± 0.053 mg of gallic acid eq./g, and it was almost constant over 9 days at room temperature (Figure 2).

The total phenolic content in Abate pears harvested on 30 September was higher (1.647 ± 0.060 mg of gallic acid eq./g), thus confirming the literature evidence that ripening and the harvesting period can affect the product characteristics. On the other hand, the polyphenol content after cold storage was stable for two months and also for the 9 days at room temperature. The highest phenol content was detected in Abate fruits treated with 1-MCP and left at room temperature for 3 days (Figure 2).

Our results highlighted that 1-MCP treatment positively affected the phenolic content. In this context, Park et al. [48] and Zhang et al. [49] indicated that phenolic accumulation was related to ethylene signaling, and 1-MCP (an ethylene inhibitor) treatment might stimulate flavonoid biosynthesis and thus promote flavonoid and phenolic accumulation by regulating gene expression and enzyme activity. Moreover, phenolics with one or more hydroxyl groups exhibit antioxidant activity related to reactive oxygen species scavenging and stress responses [49].

The antioxidant activity in fresh peel was estimated using a DPPH scavenging assay (Figure 2). The antioxidant activity in non-treated fruits harvested on 15 September was 3.104 ± 0.049 mM of Trolox eq./g of fresh peel; products stored with 1-MCP, however, presented an increased level of antioxidant activity, which increased to 5.213 ± 0.45 mM of Trolox eq./g of fresh peel after 6 days at room temperature and decreased to 4.743 ± 0.10345 mM of Trolox eq./g of fresh peel after 9 days. As for phenolic compounds, the antioxidant activity of non-treated peel samples harvested on 30 September was higher than 15 September, confirming the importance of ripening on the chemical composition and taste as well as on phytochemical compounds. Cold storage treatment was able to maintain the antioxidant activity after 2 months. Conservation at room temperature for 3 days positively increased the antioxidant activity, while after 6 days, we detected a decrease in antioxidant activity.

As evidenced by numerous works in the scientific literature, many substances play important roles in the antioxidant activity of food products [43,49,50,51]. Chen et al. [52] described the role of free amino acids in maintaining the high antioxidant activity of soy sauce, while Piga et al. [53] and Galvis Sanchez [25] correlated the antioxidant activity of fruits with high amounts of vitamin C.

Galvis Sanchez et al. [25] studied the polyphenol, vitamin C and antioxidant activities in six cultivars of pears in both the flesh and peel. They determined high phenol and vitamin C contents in the peel, more than in the flesh, and they concluded that the contribution of phenolic compounds to antioxidant activity in pears was significantly higher than that of vitamin C.

In our study, DPPH scavenging assay values were positively correlated with the phenolic content for both the peels of samples treated with 1-MCP and peels of samples simply cold-stored, with a correlation of r = 0.8835 and r = 0.8689, respectively, confirming that as the polyphenol content increased, the antioxidant activity of pear peels increased.

It was noted that SmartFresh^®^ treatment during storage preserved the phenolic compounds and antioxidant activity of pear fruits slightly better than cold storage, and the high amounts of these phytochemicals during long-term storage also seemed to be linked to a reduction in superficial scalds, the most common physiological disorder in pears, which can cause huge economic losses and reduce the product quality [46,54,55].

**Figure 3:**
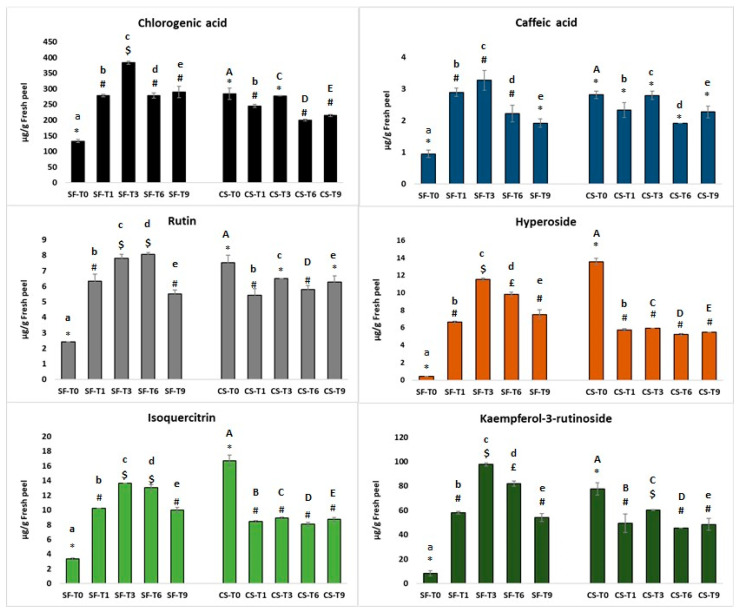
Chlorogenic and caffeic acid, rutin, hyperoside, kaempferol-3-rutinoside and isoquercitrin quantified in Abate Fétel peels harvested at two different points and stored at low temperature and with 1-MCP. The results were expressed as means ± standard deviations (µg/g of fresh matter). Different symbols indicate significant differences between samples of the same storage treatment. Lower- and uppercase letters indicate significant differences for SmartFresh (SF) samples vs. cold storage (CS) samples at the same sampling time (T0, T1, T3, T6, T9) (*p* ≤ 0.05).

Taking into consideration the high correlation between antioxidant activity and polyphenol content and the positive effect of treatment on these compounds, we have detected and identified the phenolic composition of Abate Fétel pear fruit peels (Figure 3).

Several classes of phenolic compounds in different pear cultivars have been identified [45,46,56] as associated with resistance to scald development and preserving shelf-life, and Kolniak-Ostek et al. highlighted that in “Radana” pears, the main polyphenols present were caffeic acid and its derivatives and monomeric catechins [36]. We detected and quantified six phenolic compounds via standard curve calibrations (Figure 3): chlorogenic (Rt = 6.17 min) and caffeic acids (Rt = 8.48 min), rutin (Rt = 15.20 min), hyperoside (Rt = 15.98 min), isoquercitrin (Rt = 16.28 min) and kaempferol-3-rutinoside (Rt = 18.4 min). These compounds were identified and quantified in both samples stored with 1-MCP treatment and cold-stored samples. The most abundant compound in Abate peels was chlorogenic acid followed by kaempferol-3-rutinoside (Figure 3).

Chlorogenic acid amounts in pear peels at harvest on 15 September were the lowest, and the same trend was also noted for other phenolic compounds. At the second harvest at the end of September, however, high amounts of each polyphenol were detected.

Regarding storage with 1-MCP, all phenol compounds increased during the two months and reached a maximum after 3 days of storage at room temperature and then decreased after 6 days. This was except for rutin, which was also present in high amounts after 6 days of storage at room temperature. Despite the decrease in polyphenol content, after 9 days of storage, the values of each compound were still high and comparable with sample T0.

Abate Fétel pears harvested on 30 September exhibited a higher quantity of each phenolic compound than the samples from 15 September; this may be due to riper fruits. Fruits undergoing the cold storage treatment exhibited a constant amount of chlorogenic and caffeic acids after two months of storage in climatic cells, while rutin, hyperoside, kaempferol-3-rutinoside and isoquercitrin amounts decreased. Rutin showed a loss of about 30%, kaempferol-3-rutinoside a loss of 35%, while isoquercitrin and hyperoside showed 50% and 58%, respectively.

A comparison of SmartFresh^®^ and cold treatments showed that 1-MCP efficiently conserved phenol compounds in fruit peels, with higher amounts of all detected polyphenols than cold-stored samples. The results clearly showed that 1-MCP treatment increased the total amount of phenols, guaranteeing a good fruit shelf-life. Our results were supported by results reported by other authors [57,58,59], who demonstrated the 1-MCP treatment stimulated polyphenol biosynthesis, and specifically [49] 1-MCP treatment increased the content of phenolic acids upregulating gene expression and retarded phenolics degradation by suppressing peroxidase and polyphenol oxidase activity, delaying senescence and extending the fruit shelf-life.

Most studies on the chemical composition and health-promoting compounds of pears with different storage treatments were focused on peeled fruits, although most of the active components are mainly present in the fruit peel. Although pear fruits are presumed to be consumed fresh or minimally processed, this fruit is also commonly found in processed products such as baby foods, drinks, marmalades, preserved products and jams. Some of the industrial processes to produce these products lead to the production of by-products such as peels, which are rich in antioxidant compounds important for functional foods and the nutraceutical industry.

At the same time, given the increasing attention towards organic pesticides and natural products in agriculture, the use of fruits with a peel is increasingly recommended, not only for their high fiber content but also for the high amounts of phytochemical compounds. These compounds provide a synergistic and cumulative effect on protecting human health and disease prevention. Therefore, it is possible to affirm that 1-MCP treatment is able to extend the shelf-life of fruits, maintaining the pulp aroma and taste and the active compounds in peels in addition to reducing superficial scalds. Meanwhile, cold treatment was less efficient, especially in conserving antioxidant compounds.

## 4. Conclusions

The present study provides information regarding the use of 1-MCP for Abate Fétel pears in comparison with the more classic storage in a cold room at refrigerator temperatures. The study was conducted on pear fruits harvested at the stage of agronomic ripeness but at two different times spaced 15 days apart. We evaluated some important characteristics of the pulp, highly associated with the delicate and pleasant aroma and flavor of this fruit. In addition, the glucose and fructose concentrations, the pH, the acidity, and the organic acid contents were determined. Sugar contents and pH levels did not change between the two harvesting times and were not influenced by the treatment used. The acidity, expressed in terms of malic acid, was significantly lower in pears harvested at the end of September, but sugars were preserved in both treatments. Due to the lower acidity at the second harvest, the sugar/acidity ratio was higher in samples collected on 30 September and was maintained during the storage period.

The main organic acids found in the fruits were malic and shikimic acids, followed by citric and fumaric acids. The former three organic acids were conserved in both storage treatments, but the fumaric acid contents decreased, especially after 1-MCP treatment. Furthermore, apart from the concentration in malic acid, which was found to be higher at the first harvest, the concentrations in organic acids were not significantly different between the two collection times, even following respective treatments. The phenolic compounds and antioxidant activities were analyzed, with attention being focused on pear peels. The peel was richest in these compounds, which is of particular interest for extending the shelf-life of fruits but also for the extraction of phenolic components to use as “natural antioxidants” in the nutraceutical industry, and this is no less important for the consumption of the whole, fresh fruit. Our data highlighted that the samples harvested on 15 September had the lowest amounts of phenolic compounds and a lower antioxidant activity than the samples harvested on 30 September, but after 2 months of conservation, the phenolics and antioxidants presented the same concentrations regardless of the preservation method or harvest time. Moreover, 1-MCP treatment conserved antioxidant compounds more than refrigeration, preserving the phenolic contents and high antioxidant activity even with an early harvest. Hence, in this study, we demonstrated that Italian Abate Fétel pears are an interesting source of active compounds with antioxidant activity; 1-MCP treatment and cold storage can be considered two effective storage methods with which to preserve the flesh and peel composition and guarantee a long shelf-life and high phytochemical amounts, even in fruits harvested a little time apart, which is a normal agronomic practice.

## Figures and Tables

**Table 1:** Glucose, fructose, pH and acidity in cold-stored pear samples and stored after SmartFresh^®^ treatment (1-MCP), harvested in two different times. All data are expressed as fresh matter, glucose and fructose are expressed as g/100 g of fresh matter, and acidity is expressed as g of malic acid equivalent/100 g of fresh matter. Values are means ± standard deviation (SD); *n* = 3. Different symbols indicate significant differences between samples of the same storage treatment. Lower- and uppercase letters indicate significant differences for SmartFresh (SF) samples vs. cold storage (CS) samples at the same sampling times (T0, T1, T3, T6, T9) (*p* ≤ 0.05).

Sample	Glucose (g/100 g)	Fructose (g/100 g)	pH	Acidity (Malic Acid eq./100 g)	Ratio Sugar/Acidity
SF-T0	1.95 ± 0.04 *a	5.30 ± 0.07 *a	4.64 ± 0.02 *a	1.28 ± 0.01 *a	5.68 *a
SF-T1	4.92 ± 0.09 #b	8.54 ± 0.05 #b	4.71 ± 0.02 *b	1.24 ± 0.01 *b	10.90 #b
SF-T3	5.63 ± 0.12 $c	8.03 ± 0.23 $c	4.79 ± 0.03 #c	1.16 ± 0.00 #c	11.78 #c
SF-T6	5.32 ± 0.12 $d	8.29 ± 0.23 #d	4.79 ± 0.05 #d	1.17 ± 0.03 #d	11.63 #d
SF-T9	5.55 ± 0.10 $e	8.01 ± 0.09 $e	4.68 ± 0.04 *e	1.15 ± 0.02 #e	11.84 #e
CS-T0	2.53 ± 0.01 *a	5.25 ± 0.07 *a	4.66 ± 0.01 *a	1.00 ± 0.05 *A	7.81 *A
CS-T1	5.72 ± 0.33 #B	8.24 ± 0.13 #b	4.96 ± 0.01 #B	0.87 ± 0.00 #B	16.04 #B
CS-T3	5.71 ± 0.10 # c	8.28 ± 0.05 #c	4.90 ± 0.02 #c	1.03 ± 0.01 *C	13.32 $C
CS-T6	6.07 ± 0.14 #D	8.35 ± 0.15 #d	4.84 ± 0.05 #d	1.07 ± 0.03 *D	13.48 $D
CS-T9	5.71 ± 0.02 #e	8.49 ± 0.11 #E	4.13 ± 0.04 $E	1.06 ± 0.01 *E	13.46 $E

**Table 2:** Effects of 1-MCP and cold storage on fumaric, malic, citric and shikimic acid contents during storage of Abate Fétel fruits harvested at two different times. All data are expressed as fresh matter. Values are means ± standard deviation (SD); *n* = 3. Different symbols indicate significant differences between samples of the same storage treatment. Lower- and uppercase letters indicate significant differences for SmartFresh (SF) samples vs. cold storage (CS) samples at the same sampling time (T0, T1, T3, T6, T9) (*p* ≤ 0.05).

Sample	Fumaric Acid (µg/g)	Malic Acid (µg/g)	Citric Acid (µg/g)	Shikimic Acid (µg/g)
SF-T0	5.04 ± 0.04 *a	2804.49 ± 7.48 *a	34.49 ± 1.82 *a	116.53 ± 1.03 *a
SF-T1	3.90 ± 0.40 *b	1989.70 ± 161.20 *b	21.60 ± 2.70 *b	113.10 ± 8.50 *b
SF-T3	1.60 ± 0.10 #c	2372.70 ± 164.90 *c	38.90 ± 2.10 *c	85.60 ± 2.00 *c
SF-T6	0.90 ± 0.10 #d	2075.00 ± 205.60 *d	40.80 ± 4.60 #d	83.40 ± 2.60 *d
SF-T9	1.00 ± 0.30 #e	1868.60 ± 64.90 *e	43.90 ± 3.60 #e	83.70 ± 3.20 *e
CS-T0	5.09 ± 0.01 *a	2025.63 ± 7.96 *A	46.71 ± 0.86 *a	103.70 ± 1.55 *a
CS-T1	3.80 ± 0.50 *b	1813.10 ± 17.00 *b	36.30 ± 2.40 *b	103.80 ± 1.50 *b
CS-T3	3.10 ± 0.50 #C	2092.80 ± 180.20 *c	48.70 ± 1.40 *c	106.30 ± 9.40 *c
CS-T6	2.40 ± 0.30 #D	1809.50 ± 45.90 *d	50.00 ± 4.70 *d	100.90 ± 0.80 *d
CS-T9	1.30 ± 0.10 $e	1922.30 ± 128.40 *e	52.40 ± 5.20 *e	83.40 ± 3.40 *e

## Data Availability

Not applicable.
